# Notes on Preparations for the Sick

**Published:** 1911-09

**Authors:** 


### Notes on Preparations for the Sick.

**British Medical Association, Birmingham.**—An attractive feature of the exhibit of Burroughs, Wellcome & Co. was the fine collection of medicinal plants grown at the "Wellcome" Materia Medica Farm. These plants are raised under ideal conditions, and their cultivation near the "Wellcome" Chemical Works and under the superintendence of experts has resulted in the selection and maintenance of the strains of each plant best for medicinal purposes.

**"Tabloid" "Xaxa" and Caffeine** is issued by Burroughs, Wellcome & Co., containing 4 grains of "Xaxa" (acetyl-salicylic acid) and a grain of caffeine. It combines the specific antipyretic and analgesic action of "Xaxa" with the stimulant effect of caffeine on the circulation. It should, therefore, be of value in cases of pyrexia where the heart's action is weak. The diuretic action of the caffeine is also useful in febrile conditions. The dose is from one to five twice or thrice daily.

**"Epinine,"** the remarkable synthetic vaso-constrictor and hæmostatic recently introduced by Burroughs, Wellcome & Co., has now been issued in combination with cocaine hydrochloride. The dose for hypodermic injection for dental purpose is 1 c.c.

**Junora.**—HUMPHREY, TAYLOR & CO., 45 New Oxford Street, London.—A light, dry wine containing about the same amount of alcohol as a light claret, and an efficient quantity of Lecithin-ovo. The value of the organic glycero-phosphates in the constructive metabolism of the nervous system and of the red corpuscles is well recognised, and this combination with a palatable light wine should be valuable to a large class of invalids and persons who are doing brain work which calls for some nerve stimulant.

We have tested the wine, and found that it appeared to have an invigorating effect, which did much to counteract the exhausting effects of hot weather.

**Dr. Voebt's Legumine Biscuits.**—WILCOX, JOZEAU & CO., London.—Many attempts have been made to devise a satisfactory substitute for bread, and yet at the same time to provide a palatable, easily digested, nutritious food. The difficulty has been overcome in Voebt's Biscuits by converting the starch into erythrodextrine by means of an excess of diastase, the product is then dried and mixed with legumine or vegetable albumin, this being selected on account of its richness in nitrogen and phosphorus. Test meals prove them to be entirely soluble and readily digested, no trace being found twenty minutes after ingestion. They increase the activity of gastric digestion by stimulating, but not exhausting, the secretion from the gastric glands, with rapid formation of peptones. No fermentation takes place, so leucomains or other toxic products cannot be formed. The biscuits were originally manufactured to supply a long-felt want of a diet suitable for the use of diabetic patients, which was palatable and nutritious, and which the patient would not tire of. They have since been found of great use in all forms of dyspepsia, gastritis, gastralgia, dilatation and atony of the stomach, enteritis, chronic and dysenteric diarrhœa; in fact, whenever it is desirable to have a minimum of solid residue. After convalescence from operations, particularly abdominal ones, after fever and acute illness, their power to build up the muscular system renders them a most appropriate item in a hygienic diet, and at all times when there is a tendency to undue fat formation or to put on weight.

